# Agentic AI workflows in anatomic pathology: Opportunities and challenges

**DOI:** 10.1016/j.jpi.2026.100718

**Published:** 2026-09-12

**Authors:** Andy Ngo, Roland Tian, Amin Mohammad, Jenny L. Weon, Simone Arvisais-Anhalt, Jacob Minor, Kenneth Youens

**Affiliations:** a Clinical Informatics Fellowship Program, Baylor Scott & White Health, Temple, TX, United States of America; b Department of Pathology, Baylor Scott & White Health, Temple, TX, United States of America; c Department of Pathology, UT Southwestern Medical Center, Dallas, TX, United States of America; d Department of Laboratory Medicine, University of California, San Francisco, San Francisco, CA, United States of America

**Keywords:** Large language models, Anatomic pathology, Agentic AI, Retrieval-augmented generation, Clinical decision support, Workflow automation, Lab information systems, Pathology reporting, Human-in-the-loop, Artificial intelligence

## Abstract

Artificial intelligence (AI) in anatomic pathology is dominated by image-based diagnostic applications, but much of the specialty's cognitive work is textual: reviewing clinical history, drafting reports, coordinating testing, and communicating results. Large language models can be useful for many of these tasks, and agentic systems extend their capabilities by retrieving external information, invoking software tools, following task-specific instructions, tracking workflow states, and carrying out complex multi-step tasks under human oversight. This conceptual review outlines key components of agentic AI systems including retrieval augmented generation, tool use, skills, and the so-called “agentic loop”—and applies them to anatomic pathology workflows. Three illustrative design sketches use reflex testing support, workload balancing and case routing, and ancillary test decision support to explain how agentic systems can combine lab information system data, trustworthy knowledge sources, local policy, and explicit human approval gates. We examine considerations for clinical deployment, including interoperability and integration challenges, lifecycle governance and validation, security and safety concerns, and cost and sustainability. Agentic AI offers a plausible and constructive near-term vision for AI in pathology: better-supported diagnosis that allows pathologists to spend more of their time on the judgment, accountability, and genuine care that remain distinctly human.

## 1 Background

### 1.1 Introduction to LLMs

Large language models (LLMs) are now a familiar part of the information technology landscape, with products like ChatGPT (OpenAI) having among the fastest growing user bases in history.^1^ LLMs are a type of artificial intelligence (AI) system built using a machine learning architecture called a transformer. A key feature of the transformer is an attention mechanism that allows the model to assess the importance of words in a sequence relative to one another, permitting contextualization. When trained on vast amounts of data, LLMs demonstrate a remarkable ability to mimic understanding and generate human-like responses.^2^ LLMs excel at language processing tasks like document summarization, sentiment analysis, text classification, and text generation, which allows them to function remarkably well in the familiar role of a conversational chatbot.^3^

In clinical medicine, LLMs have found utility in a broad range of applications such as clinical decision support,^4^, ^5^ clinical documentation,^6^, ^7^ chart summarization,^8^ ambient listening,^9^, ^10^ and provider–patient communication.^11^, ^12^ Additional applications adjacent to direct clinical care include matching patients to clinical trials,^13^ supporting medical coding workflows,^14^ automating hospital quality-measure abstraction and reporting,^15^ drafting prior-authorization letters,^16^ and extracting clinical research data from health records.^17^ Several of these features have moved beyond the research setting and are now deployed as commercial products in active clinical use. For example, Epic Systems Corporation (Epic) offers integrated LLM functions such as note summarization, LLM-extracted findings for radiology reports, AI charting, discharge summary drafting, encounter coding, patient-message responses, end-of-shift nursing notes, electronic prior authorization responses, and other clinician-facing text assistance.^18^

### 1.2 Artificial intelligence in anatomic pathology

In anatomic pathology, most of the recent progress in AI has focused on diagnostic interpretive applications like tumor detection and characterization,^19^, ^20^, ^21^ quantitative immunohistochemistry (IHC) and biomarker scoring,^22^ quality assurance and whole-slide image quality control,^23^, ^24^ and prognostication or treatment–response prediction, including models that combine digital pathology image features with clinical variables.^25^ At the time of this writing, a few anatomic pathology AI products have received FDA authorization, including diagnostic support tools like Paige Prostate^26^ and Ibex Galen Second Read,^27^ prognostic/predictive models like ArteraAI prostate,^28^ and cytology systems like the Genius Cervical AI algorithm,^29^ with more in the industry pipeline.

However, these diagnostic and prognostic applications address only one facet of pathology practice. Much, if not most, of the specialty's cognitive work occurs in language form, not image form—reviewing clinical history, writing diagnostic reports, coordinating ancillary testing, working with local lab teams, and communicating results. These language-heavy, often document-based tasks, combined with the relatively predictable workflows of anatomic pathology, make anatomic pathology amenable to LLM integrations. Various pathology-related LLM applications have been conceived, such as structured extraction of diagnostic information from free-text histopathology reports,^30^, ^31^, ^32^, ^33^, ^34^ automated synoptic-report generation from narrative cancer pathology reports,^35^ summarization of complex longitudinal cancer pathology reports for clinical usability,^36^ error detection in pathology reports,^37^ pathology-related knowledge support,^38^ pathology report generation,^39^ support for coding or classification workflows based on pathology report text,^40^ and evaluation of pathology-related image and textual inputs for question answering.^41^

### 1.3 Agentic workflows

The language-heavy examples above use LLMs to read, draft, summarize, classify, proofread, and otherwise manipulate pathology-related text. But what if, in addition to manipulating text, the LLM could take action on our behalf? Rather than simply manipulating some text or responding to a prompt, the LLM could actively participate in the work by making decisions, coordinating tasks, remembering preferences, and taking actions autonomously, routing work back to a human only when judgment or authorization is required. Such an arrangement, where an LLM is given access to external tools, data, and instruction sets to complete multi-step tasks, is often called an “agentic” system. The term “agent” has a long history in AI, where it generally refers to a system that can act within an environment rather than merely classify an input or generate a static output.^42^ In the current LLM literature, “agentic” systems are usually described as LLM-based systems that can reason over a task, interact with tools or other systems, and proceed through multiple steps toward a goal.^43^, ^44^, ^45^, ^46^ Radiology reviews have framed agentic systems as a shift from passive, user-triggered LLM outputs toward systems that can interact with tools and data sources, coordinate workflow steps, and support clinical work.^47^, ^48^, ^49^ As described above, the pathology literature describes a broad array of generative AI applications, including report generation, information extraction, quality control, workflow support, and multi-modal systems, and describes incorporation of these capabilities into agentic workflows.^50^, ^51^, ^52^ Several pathology-related agentic systems or component tools have been developed and evaluated, including LLM-based structured extraction from pathology reports,^32^ agentic orchestration of medical-image analysis,^53^ agentic generation, coding, and retrospective evaluation of computational pathology hypotheses,^54^ and multi-agent rare-disease differential-diagnosis support.^55^ For purposes of this article, an agentic workflow refers to a workflow in which one or more AI “agents” use an LLM, external tools, institutional data, and predefined instruction sets to complete multi-step anatomic pathology tasks under human oversight but with little direct human intervention.

In this article, we provide concise technical background, and present a summary and examples of potential applications of agentic AI for non-diagnostic applications in anatomic pathology. Further, we provide commentary on the potential opportunities and barriers to the deployment of agentic AI in a clinical anatomic pathology setting.

## 2 Technical primer

### 2.1 Basic LLM functionality

At the simplest level, an LLM can be used in a one-shot fashion, whereby a user gives the model a single prompt, and the model returns a single text output (Fig. 1). The prompt may include a question, some text to summarize, useful contextual information, example responses, formatting instructions, a description of constraints on the desired answer, or any combination of these or other inputs. The term “prompt engineering” refers to the practice of deliberately designing these inputs so the model receives clearer instructions, better context, examples when useful, and more explicit output constraints, all of which can improve the quality and usefulness of model outputs.^56^

**Fig. 1:**
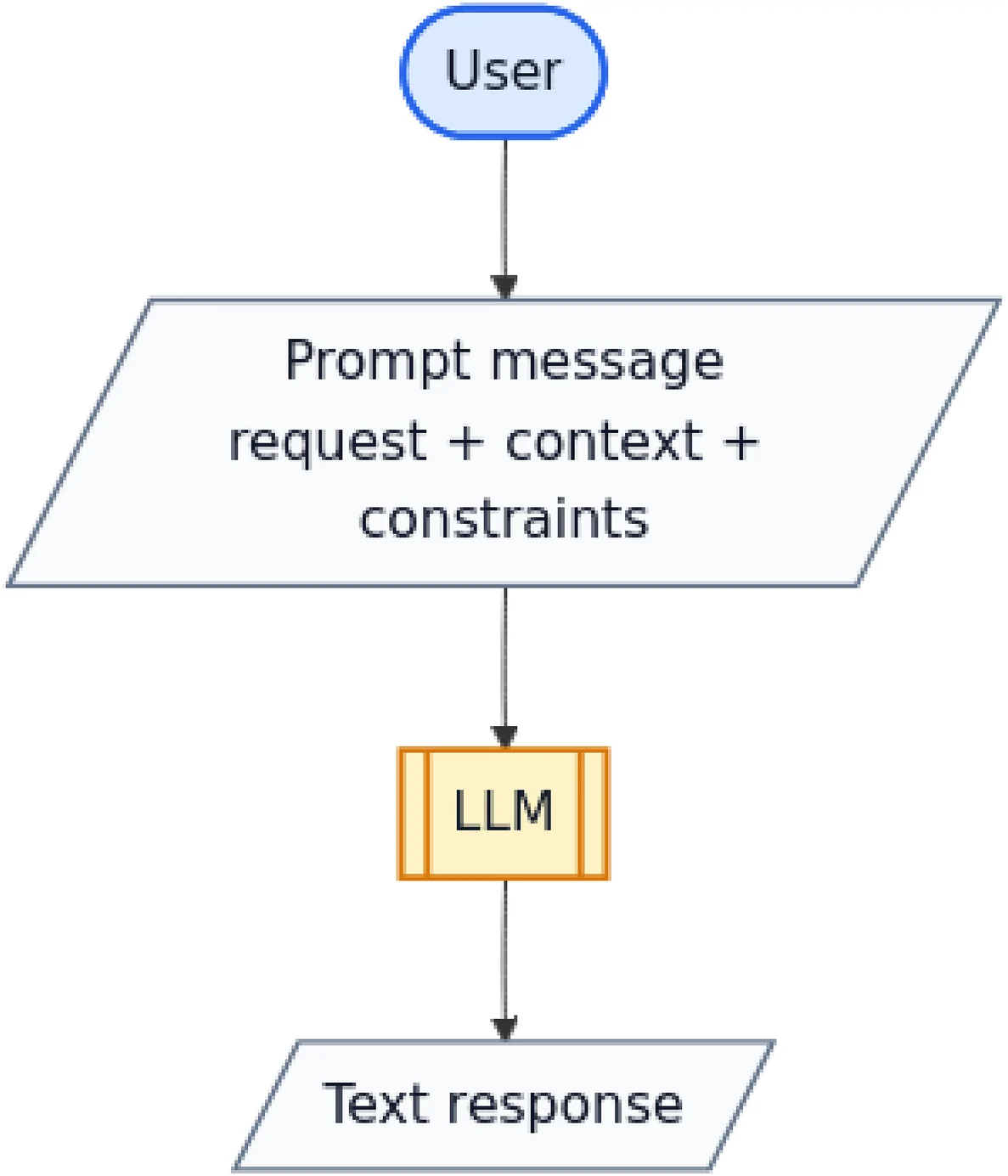
*One-shot LLM interaction pattern.* A user supplies a single prompt message, and the LLM generates a single text response.

A chatbot extends this basic pattern by repeating the exchange over a series of messages. Instead of receiving only a single prompt, the model receives the current user message along with prior conversation context, generates the next response, and waits for another input by the user (Fig. 2). This basic prompt–response pattern is the foundation for familiar consumer applications such as ChatGPT and Claude.

**Fig. 2:**
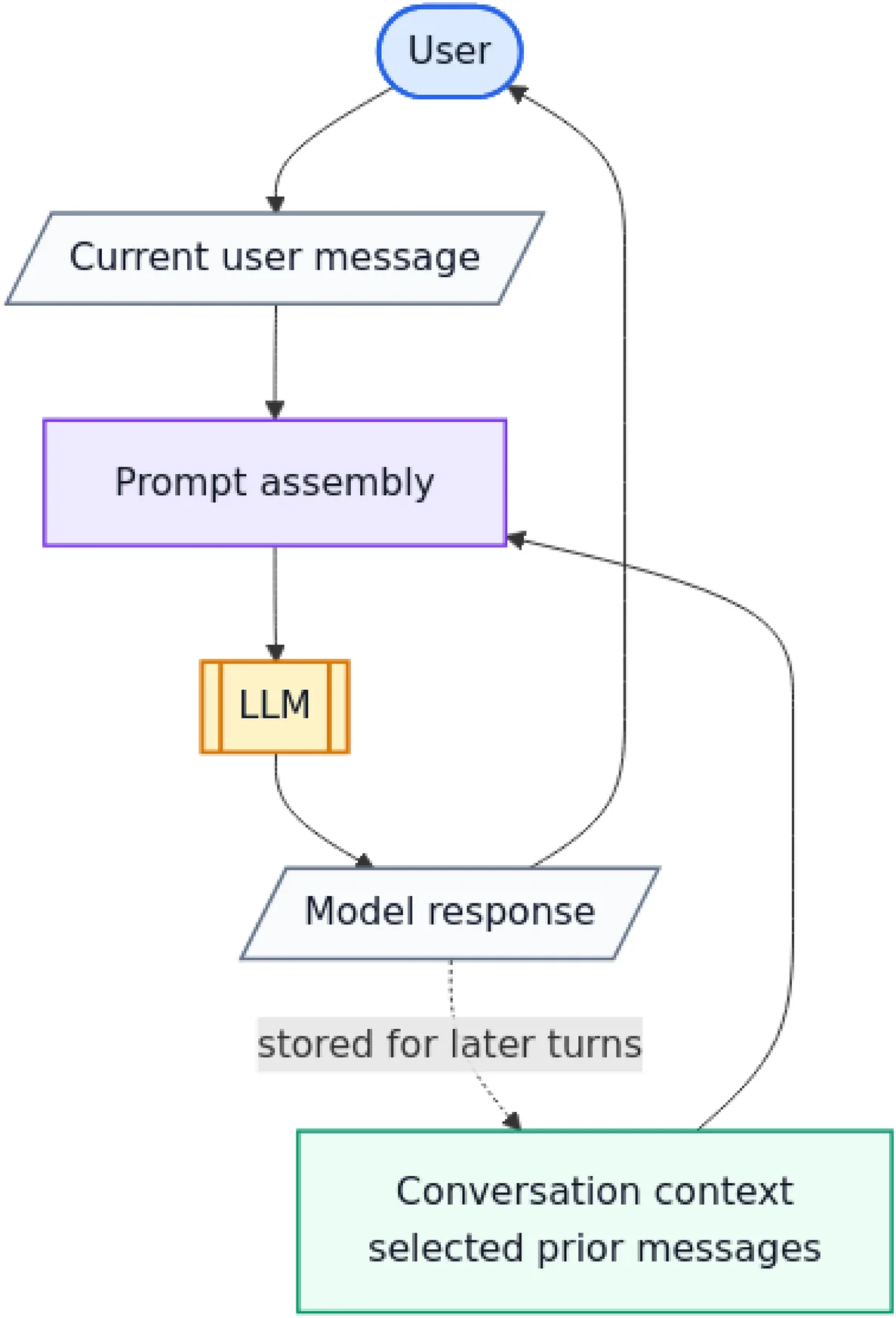
*Chatbot interaction pattern.* A current user message and prior conversation context are assembled into the input sent to the LLM, which generates the next model response.

The more advanced LLM design patterns described below build on the same prompt–response foundation, but add retrieval, tools, memory, planning, and execution layers, so that the model can work with institution-specific information and participate in multi-step processes rather than simply responding to individual prompts.

### 2.2 Advanced LLM-related design patterns

The basic LLM design patterns described above can be extended by making software tools available to the models. Much of the practical utility of modern LLM systems comes not from the model alone, but from the software tooling built around it. A few of these design patterns are worth reviewing to build an understanding of how agentic AI can function.

#### 2.2.1 Retrieval-augmented generation

LLMs are remarkably capable with language, but an LLM by itself is limited to the information in its training data and whatever context is placed into the prompt at the time of use. With retrieval augmented generation (RAG), the LLM is supplied with information retrieved at the time of the request, rather than relying only on information that the model inherently “knows” from its training data (Fig. 3).^57^ The retrieved information can come from any accessible external data source. For example, in an anatomic pathology context, a RAG system might retrieve prior surgical pathology reports, relevant clinical history, synoptic reporting rules, or institutional reflex testing policies, and supply that up-to-date local information to the model to inform its response. Critically, RAG permits tracing of the provenance of model outputs. Unlike a standalone LLM response, RAG-supported responses can expose the original source documents, textual passages, reports, or data elements that were retrieved and used to generate the answer. This makes the output amenable to review, validation, and audit, which are especially important in healthcare settings where explainability, accuracy, and accountability are requirements.^58^ RAG is also well-suited to rapidly evolving knowledge sets, because updating an external data source is easier, faster, and cheaper than retraining or fine-tuning the underlying model.^59^ RAG systems can also reduce model errors when the LLM is confronted with queries about highly specialized knowledge, though they do not eliminate these errors entirely.^60^ The role of a RAG system in the broader agentic architecture is to provide traceable, timely information to support downstream LLM outputs.

**Fig. 3:**
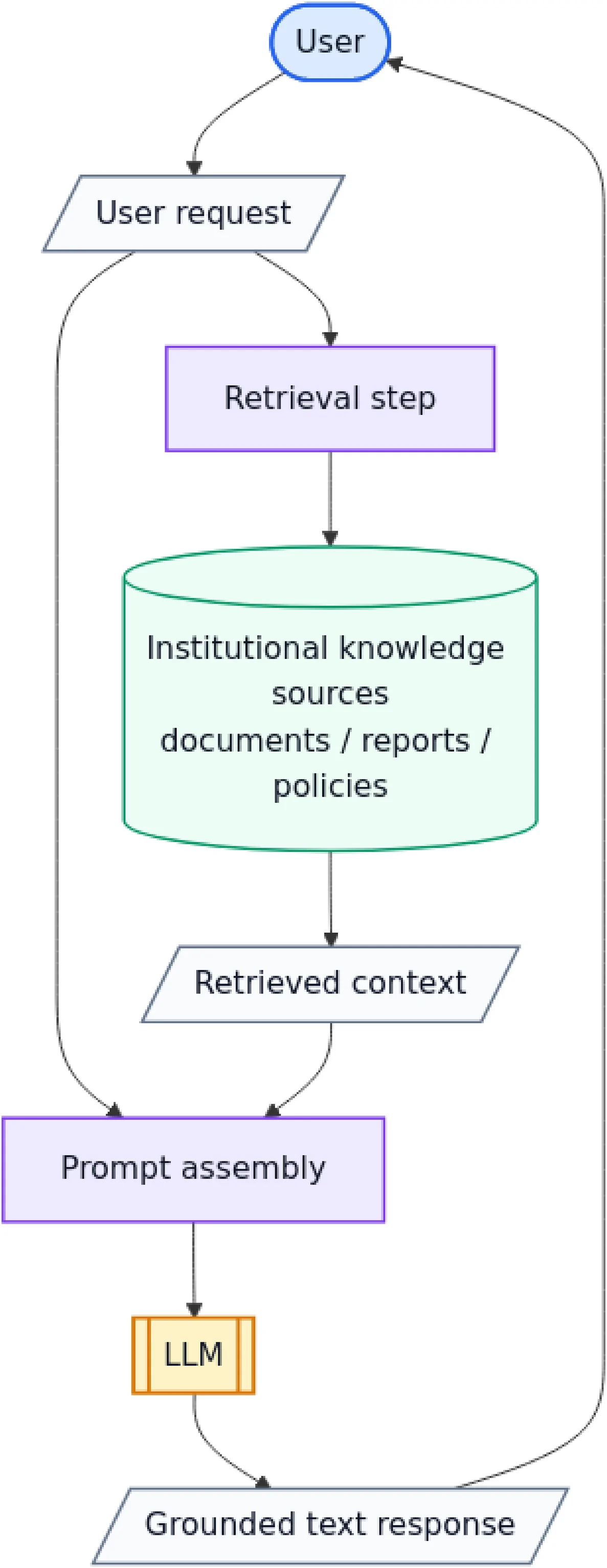
*Retrieval-augmented generation pattern.* A user request is used to retrieve relevant institutional context before prompt assembly and LLM response generation.

#### 2.2.2 Tool use

Whereas RAG gives the model access to traceable and timely information, tool use gives the model access to external capabilities and the ability to take action. In a basic tool use workflow, an LLM uses a piece of external software (a “tool”) to perform a specific function, receives the result, and then uses the result in the next step in generation (Fig. 4). The tool itself can be almost any software capability. A tool could query a database, search a document repository, run a command-line utility, or use an application programming interface (API) to interact with a server in order to take an action. In an anatomic pathology workflow, for example, a model with tool access to the laboratory information system (LIS) could retrieve prior accessions for the same patient, place orders for special stains, proofread draft report contents, and assemble a draft follow-up message about a significant diagnosis.

**Fig. 4:**
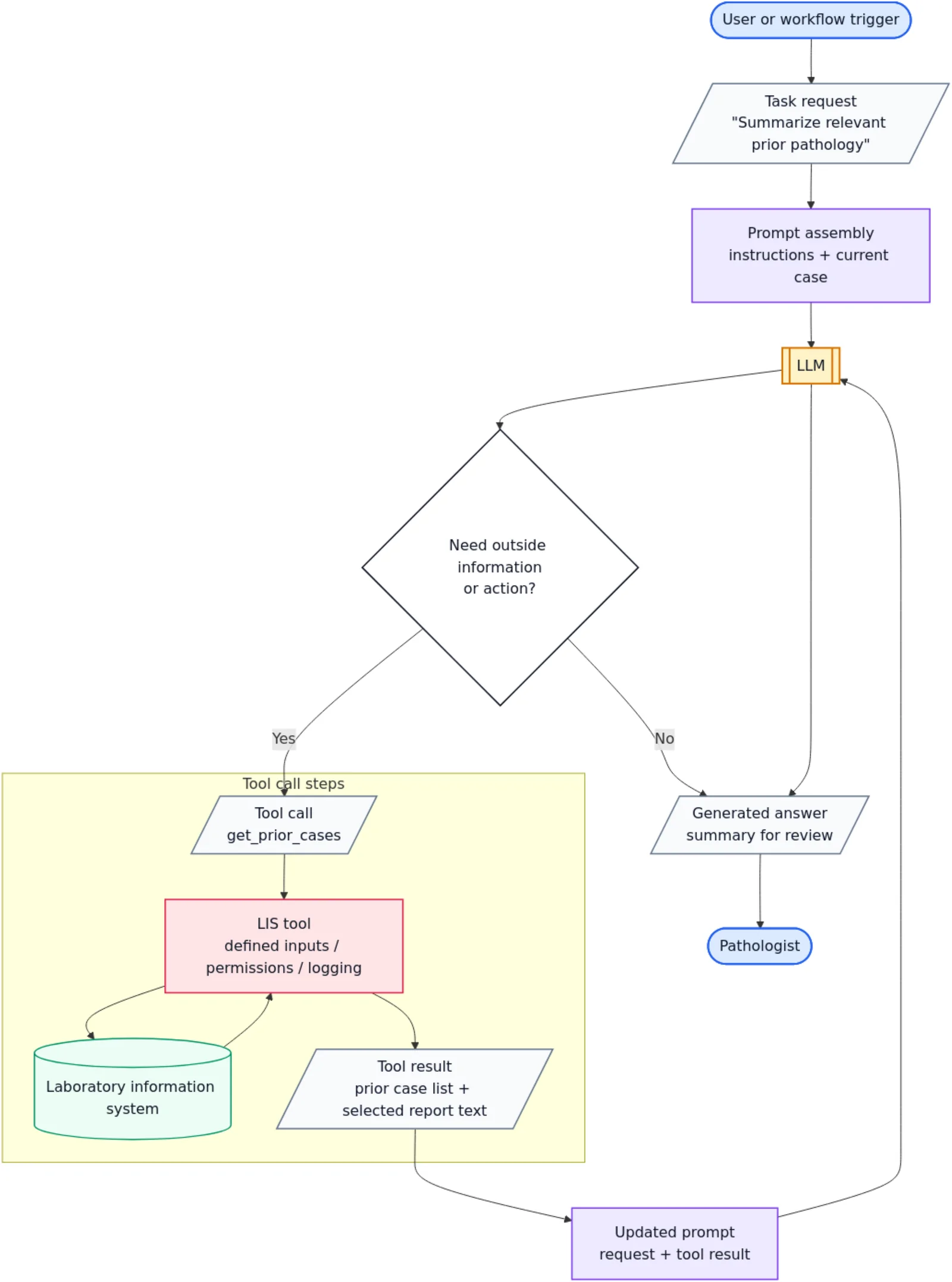
*Tool-use pattern.* In an example pathology workflow, the model might call a read-only LIS tool that returns a limited set of prior pathology reports for the current case, then use those returned reports to draft a concise summary for pathologist review. The LLM uses a tool call, shown in the yellow box, to get previous case information from the LIS. The returned tool output is then added back into the prompt context before the model generates a response for pathologist review. (For interpretation of the references to color in this figure legend, the reader is referred to the web version of this article.)

Tool access can be implemented in several ways. Some software exposes functionality to LLMs through APIs or internal service endpoints. Others wrap command-line programs, database queries, search indexes, or application-specific functions behind standardized interface patterns usable by LLMs. For example, protocols such as the model context protocol (MCP) have attempted to standardize how models discover and use external resources and tools.^61^, ^62^

Regardless of the protocol used, the model is not typically given unlimited access to external systems. Instead, it is allowed to invoke specific functions with defined inputs, outputs, permissions, and logging. These constraints allow useful capabilities to be made available to the model while preserving boundaries, auditability, and operational controls. For example, a tool call might be limited to read-only access, restricted to a particular patient or accession context, routed through an existing authentication system, logged for audit purposes, and held for human approval before any action that alters the medical record is taken. These safeguards are necessary because the same tools that allow a model to retrieve prior accessions, edit report contents, place orders, or calculate a workload summary could cause real-world harm if misused. In general, the potential harm is proportional to the scope of the tool's permissions.

Importantly, tool use both expands what an LLM-based system can do and transfers important aspects of AI safety and security management from the probabilistic behavioral realm of LLMs into the more familiar deterministic realm of traditional software design, access control, validation, and operational governance. In other words, rather than relying on the model to behave in a certain way based on prompt engineering, a well-configured system of tools can constrain available functions, enforce privileges, validate inputs and outputs, gate selected actions behind human approvals, and preserve an audit trail. This arrangement does not completely eliminate risk, but it does facilitate its mitigation using established software security and risk management practices.^63^

#### 2.2.3 Task-specific instruction modules

One characteristic of the tool use pattern, and of any local workflow, is that the model may not contain instructions for how to use the tool or to complete the workflow in its training set. Instead, these instructions must be defined and made available to the model on demand, so the model knows how to act. These instructions, which define the tools, information sources, prompts, constraints, and behavioral rules relevant to a particular task, are often treated as modular documents containing task instructions, sometimes termed “skills.”^64^, ^65^

A skill document is essentially a focused operating manual or behavioral program for a specific task. It might tell the model when the skill applies, what information to gather, which tools should be used, what constraints should be respected, what output format is expected, and when to escalate the work to a human. For example, a pathology history summarization skill might instruct the system to retrieve prior accessions, compare the current diagnosis with relevant prior material, draft a short continuity comment, and require pathologist review before the comment is optionally inserted into the report. A very simple skill document for this workflow is shown in Fig. 5.

**Fig. 5:**
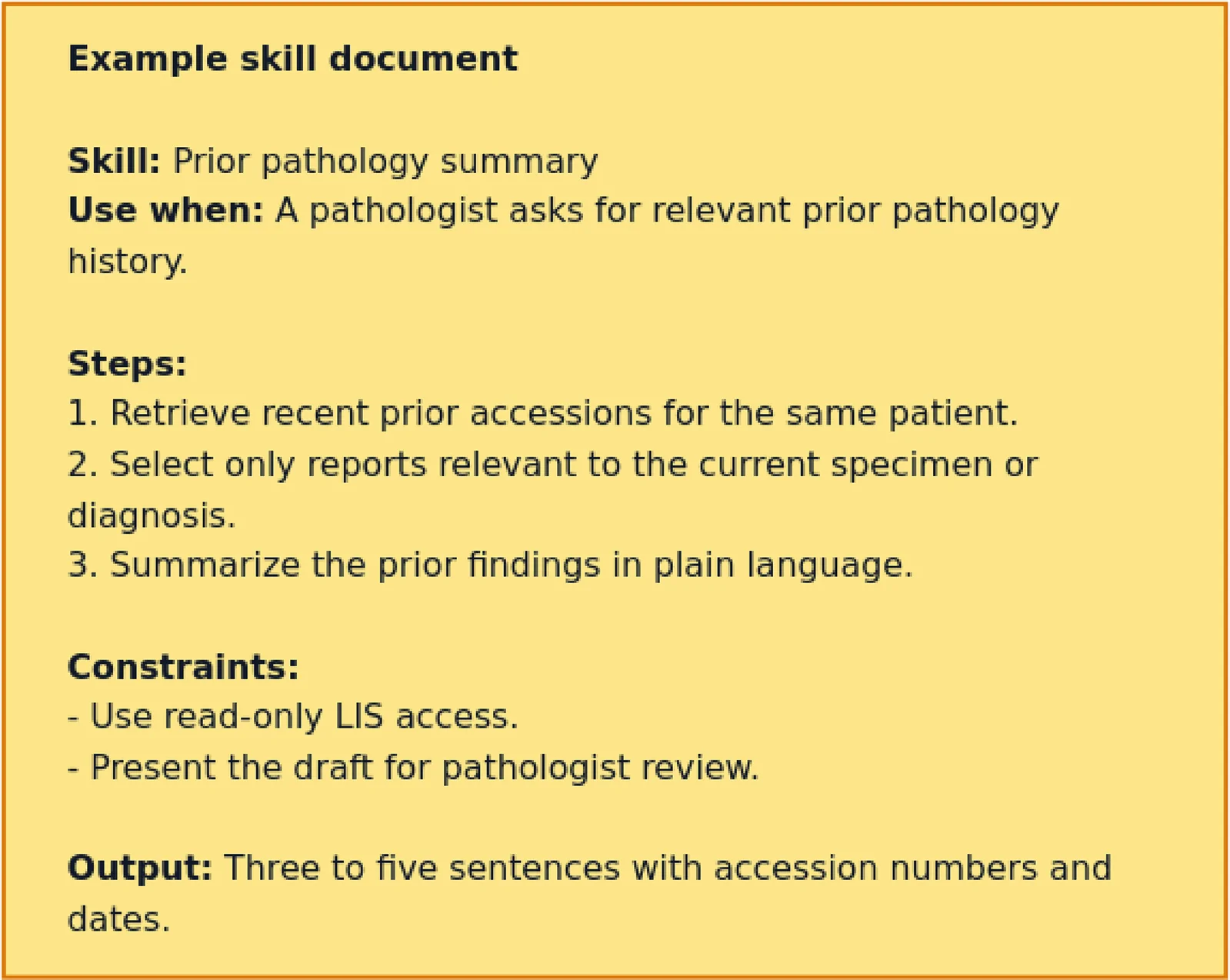
*Example skill document.* A simplified skill document for summarizing prior pathology history. In practice, a skill document gives the model task-specific instructions, boundaries, and expected output.

In use, when presented with a task, an agentic system identifies when a skill is relevant, adds the skill document to the prompt, and then lets the model proceed within that narrower framework (Fig. 6). Thus, rather than expecting a general-purpose model to know everything, an agentic system can use a system of skills to provide focused instruction sets relevant to a given task. This approach improves reliability because the model can operate within a focused, locally specified framework rather than improvising across an unlimited problem space.^66^, ^67^

**Fig. 6:**
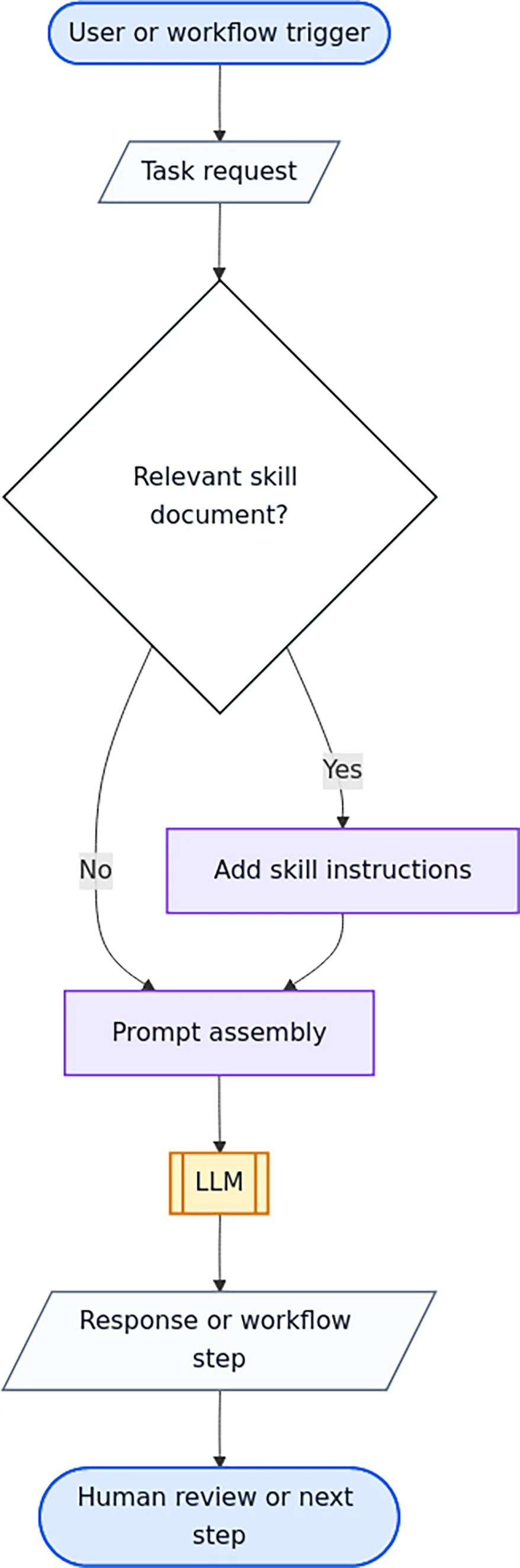
*Skill-use pattern.* The system determines whether a task-specific skill document is relevant. If so, the skill instructions are added before prompt assembly; if not, the request proceeds directly to prompt assembly.

#### 2.2.4 The agentic loop

Only a narrow subset of the cognitive work of the anatomic pathologist or lab manager is simple and linear enough to be captured in a single-prompt LLM query. A large proportion requires multi-step iterative thought processes that unfold over time: gathering, retrieving, interpreting, and synthesizing information across multiple sources, and using that information to decide what happens next. Agentic systems bind these iterative processes together using an orchestration layer that provides continuity: preserving the task state, selecting the next step, calling the relevant external module or tool, and incorporating the result before the workflow continues.^68^, ^69^ The central architectural pattern is the “agentic loop,” where the system observes and remembers the task state, asks the model to reason and plan about the next step, takes an action or retrieves additional context, incorporates the result, and repeats until the task is complete or escalation to human judgment is required (Fig. 7).

**Fig. 7:**
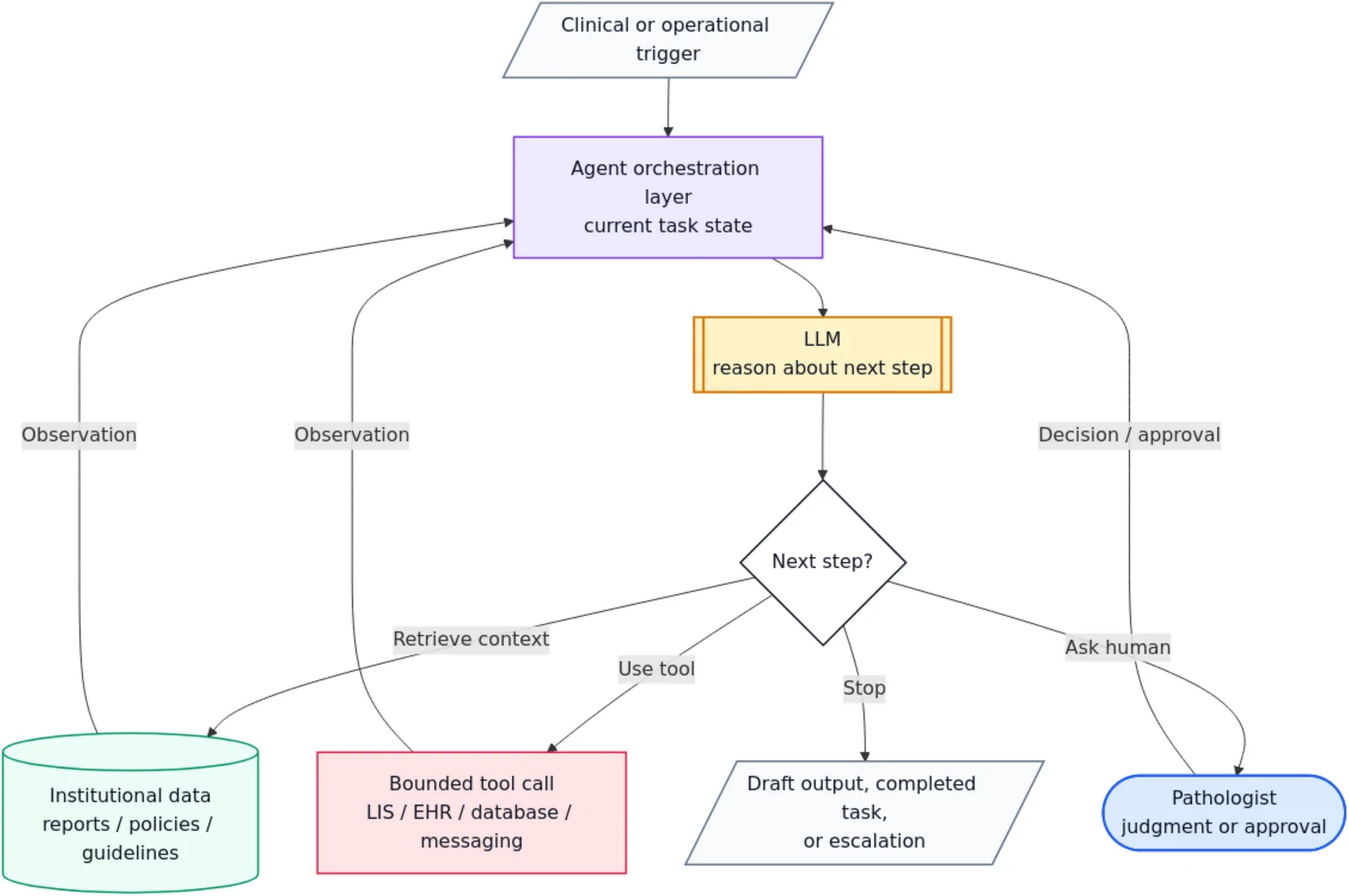
*Agentic workflow pattern.* A clinical or operational trigger engages an orchestration layer that maintains the current task state and presents the next step to the LLM. Depending on the model's response, the system may retrieve institutional data, call an external tool, request pathologist judgment or approval, or stop with a completed task, draft output, or escalation. Results from data retrieval, tool use, and human review return to the orchestration layer to inform the next step.

The ReAct (Reason+Act) framework is one well-known example of this pattern, alternating intermediate reasoning steps with actions so the model can update its plan as new observations return.^46^ This pattern is very useful when inputs are ambiguous, but sometimes model behavior can be unpredictable. So-called state-driven approaches such as StateFlow address this problem, clearly structuring the work by representing it as an explicit set of states and transitions, which can improve interpretability and consistency for multi-step tasks.^70^ Other design patterns strengthen different parts of the same loop. Some systems first break a larger task into smaller steps, then execute those steps one at a time rather than asking the model to improvise the entire workflow in a single pass.^71^ Others compare several possible next steps before choosing one, which can be useful when an early choice changes the rest of the workflow.^72^ Still others add a review step, using the model itself, a separate critic model, external feedback, or a tool-based check to decide whether the system should continue, revise the plan, stop, or escalate.^73^, ^74^ In practice, agentic systems are often hybrid architectures, combining several of these design patterns rather than relying on a single framework.^67^, ^75^ For example, a single agentic system may use a reasoning pattern to interpret an ambiguous report, and incorporate a state-driven approach to control, manage, and track which transitions and actions are allowed as downstream steps in a pathology workflow.

### 2.3 Summary

Each of these design patterns has distinct strengths and weaknesses. RAG is useful where a system requires current or locally specific source information, but RAG adds complexity and introduces the risk of retrieval failures.^57^, ^60^ Tools are necessary when the system must query data or take action, but every additional tool and set of permissions creates another opportunity for error or misuse.^63^ Task-specific instructions can encode local procedure without retraining the model, but they require revision when policies or systems change, and are susceptible to problems with semantic ambiguity or lack of precision. The agentic loop combines these elements for multi-step work, but it also allows errors to cascade and impact downstream steps. When considered together, the best system design is often the simplest possible arrangement that can complete the intended tasks safely.

### 2.4 Example technical agentic AI use cases in anatomic pathology

The following examples are simplified sketches intended to demonstrate the basic technical structures of an agentic pathology system assembled to do useful work. The main value of these examples is to illustrate two points: First, that agentic orchestration of complex workflows requiring actions like report text retrieval, prior-case lookup, RAG over curated references, incorporation of local rules, contextualization, and human approval gates is technically feasible, and second, that these examples provide the reader with a brief glimpse into the technical underpinnings of such systems. It is important to note that the provided examples are merely conceptual design sketches that have been simplified for pedagogical purposes, not depictions of deployed clinical systems. Real clinical systems would, at least, require much more comprehensive exception and error handling than is described below.

#### 2.4.1 Example 1: Reflex testing support

In this example, an institution has a reflex testing policy where every new diagnosis of colorectal carcinoma should have mismatch repair (MMR) IHC performed and reported before final verification. To enable the department's AI agent to assist with this workflow, a local subject matter expert, such as a pathology informaticist, and an LLM chatbot iteratively draft a specification for the reflex pathway in a format that is both human- and machine-readable, such as a Mermaid flowchart (Fig. 8).^76^ The agent generates a workflow template for each pathway specified in this way, and the colorectal cancer reflex rule is filed in a reflex testing skill document among many other such rules. The agent loads this skill document during case reviews, and the skill instructs the agent to compare the drafted diagnostic text to any relevant reflex rules. If the model detects a diagnosis of colorectal carcinoma, the agent is instructed to instantiate and follow a case state record from the relevant workflow template. The case state record in this example is a record that defines both the current workflow state and the case-specific metadata needed to advance the workflow according to the reflex pathway:

**Fig. 8:**
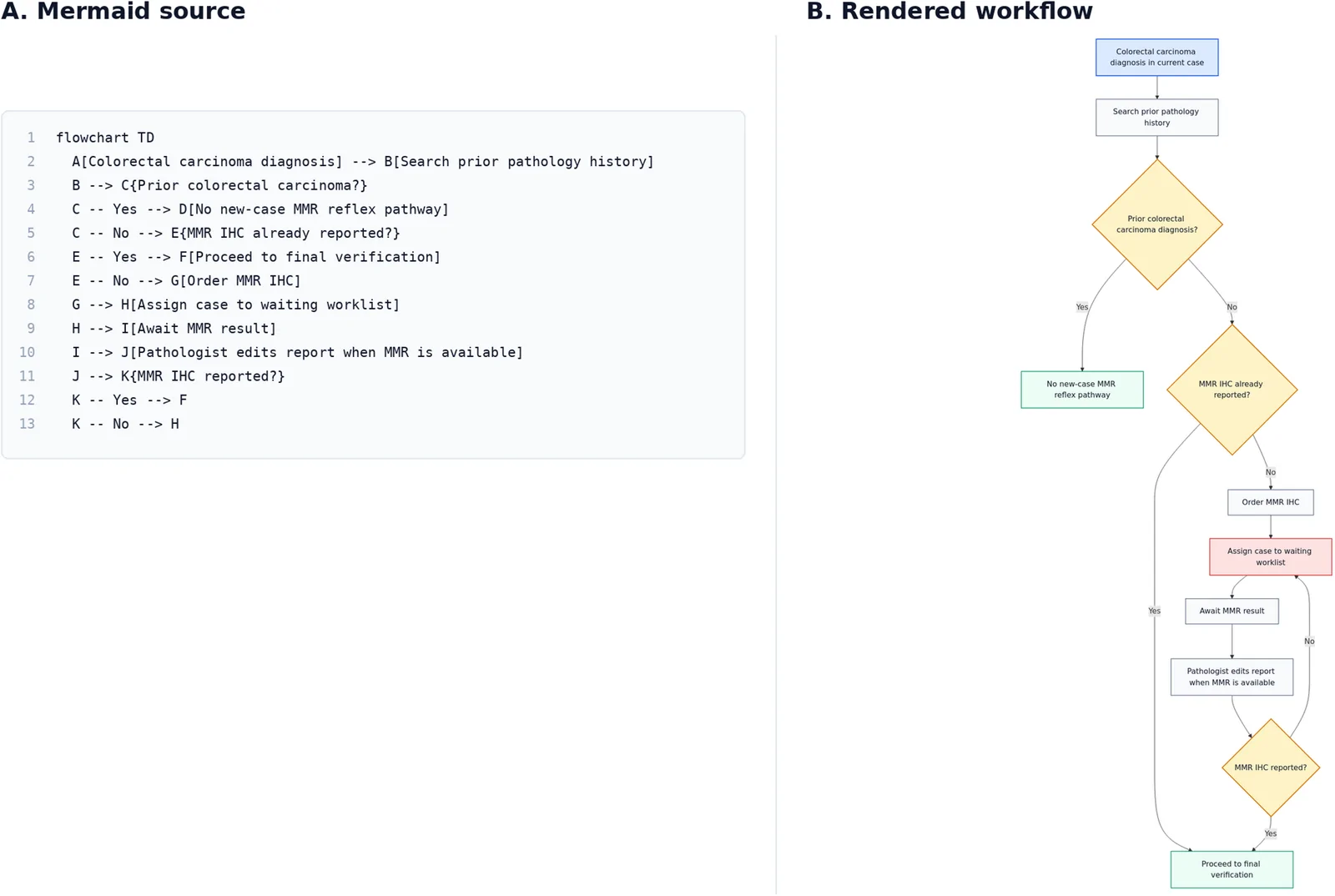
*Example MMR reflex-testing workflow.* (a) Mermaid syntax for a simplified reflex-testing workflow with a defined set of process states and transitions. (b) A flowchart-rendered workflow for a new colorectal carcinoma diagnosis. The pathway searches prior pathology history, applies the new-case reflex only when no prior colorectal carcinoma is identified, orders MMR IHC when it has not yet been reported, assigns the case to a waiting worklist, and returns the case to the pathologist for report editing when MMR is available.

{

“case_id”: “example-case-123”,

“current_state”: “search_prior_history”,

“current_case_diagnosis”: “colorectal carcinoma”,

“prior_colorectal_carcinoma”: **null**,

“mmr_ihc_reported”: **false**

}

The workflow template also defines the allowable case states and transitions. In this example, if a prior history of colorectal carcinoma is found, the reflex pathway stops; if no prior history of colorectal carcinoma is found, the agent checks whether MMR results are present in the current report draft; if they are not, the agent uses tools to order MMR IHC, and so forth:

{

“search_prior_history”: {

“prior_found”: “stop_new_case_reflex”,

“prior_not_found”: “check_mmr_reported”

},

“check_mmr_reported”: {

“reported”: “final_verification_allowed”,

“not_reported”: “order_mmr_ihc”

},

“order_mmr_ihc”: {

“order_placed”: “waiting_for_mmr”

},

“waiting_for_mmr”: {

“mmr_available”: “pathologist_report_editing”

},

“pathologist_report_editing”: {

“reported”: “final_verification_allowed”,

“not_reported”: “waiting_for_mmr”

}

}

As the agent traverses each step of the workflow, it uses relevant skills and tools for history lookup, order lookup, and stain order placement, to complete subsequent workflow steps, and uses simple scripted tools to update the case state record at each turn.

In this simple example, the agentic LLM adds value in two places. First, it helps convert a human-authored ruleset into a structured, machine-readable format. This is an attractive pattern for decision support because decision pathways are frequently revised when guidelines, local practices, or available assays change. The flowchart and downstream state definitions can be updated without rebuilding the whole system, whereas the versioned and deterministic case state record layer supports consistency.^70^, ^77^ Second, the LLM relieves the pathologist's cognitive burden by periodically checking the current case state, identifying missing information from the report, and presenting the next recommended step based on local guidelines. This basic structure is readily extensible to more complex decision support pathways, as described in the third example below.

#### 2.4.2 Example 2: Workload balancing and case routing

In the second example, an anatomic pathology practice wants to distribute incoming cases to its pathologists in a way that reflects case complexity, subspecialty expertise, scheduled service responsibilities, due dates, and accommodates unexpected interruptions. To support this workflow, the department defines a workload balancing skill that gives the agent access to tools for reading the incoming case queue, pathologist work schedule, subspecialty assignments, current case work queue status, service assignment rules, and a monitored communication channel for routing requests. The skill defines which assignments may be made automatically and which should be escalated for human review. A simplified case-routing state record might include the current assignment status, calculated workload weight, eligible pathologist pool, and escalation threshold for a batch of one or more cases:

{

“case_id”: “example-case-123”,

“current_state”: “score_incoming_cases”,

“case_queue”: “surgical_pathology_unassigned”,

“cases_pending_assignment”: 42,

“case_weight_model”: “local_complexity_weights_v1”,

“eligible_pathologist_pool”: “gi_pathology_service”,

“case_priority”: “routine”,

“requires_human_approval”: **false**,

“escalation_reason”: **null**,

“credential_requirements”: **null**

}

As in the previous example, a workflow template defines the allowed states and transitions. The agent first retrieves the unassigned case queue and applies a case-weighting scheme that has been defined locally. For example, a small GI biopsy might count as one work unit, and a complex colon cancer resection as several work units. After a batch of cases is created and the weight is assigned, the agent then retrieves pathologist schedules and current work queue assignments, excludes pathologists who are unavailable or not credentialed for the relevant service, calculates a proposed distribution based on a set of provided rules or deterministic algorithms, and either applies routine assignments or generates a message to request human approval:

{

“score_incoming_cases”: {

“scores_complete”: “retrieve_pathologist_availability”,

“scores_incomplete”: “escalate_missing_case_metadata”

},

“retrieve_pathologist_availability”: {

“availability_complete”: “propose_case_distribution”,

“availability_uncertain”: “request_human_approval”

},

“propose_case_distribution”: {

“within_routine_thresholds”: “assign_cases”,

“exception_detected”: “request_human_approval”

},

“assign_cases”: {

“assignments_written”: “monitor_routing_requests”

},

“monitor_routing_requests”: {

“routine_reroute_request”: “propose_case_distribution”,

“ambiguous_or_high_impact_request”: “request_human_approval”

},

“request_human_approval”: {

“approved”: “assign_cases”,

“rejected”: “monitor_routing_requests”

},

“escalate_missing_case_metadata”: {

“metadata_resolved”: “score_incoming_cases”

}

}

As the agent traverses this workflow, it uses several specialized tools. A queue-reading tool retrieves unassigned cases from the LIS or digital pathology system. A case-weighting tool applies the department's approved complexity model. A schedule-reading tool identifies pathologist availability, service assignments, vacations, frozen sections, tumor board obligations, and other constraints. A communication-monitoring tool watches a defined inbox or other communication channel for routing requests like “Dr. Smith has an emergency dentist appointment and will be out of the office from this afternoon. Please reassign all her urgent cases.” The agent converts those natural-language requests into structured proposed changes, checks them against workload rules, and either executes the routing change or sends a concise summary and recommendation to a supervising pathologist for review and decision.

Traditional workload management software can handle simple allocation rules, such as assigning every third gastrointestinal biopsy to the next available pathologist or routing certain kinds of cases to a fixed subspecialty pool. Deterministic rules work well when inputs are clean and rules are fixed, but it is difficult to model every unstructured and sometimes chaotic workload change that occurs throughout the day: a case arrives late, someone is pulled into an emergency meeting, someone has to leave the hospital unexpectedly, or someone asks to reroute a case through email. In this respect, an agentic system is more powerful than a purely algorithmic case assignment system. It can maintain a live representation of the workload state, retrieve missing context or recognize when clarification is needed, interpret natural-language requests, and decide whether a requested change fits within an approved routing policy. The agent's primary value is in mediating between chaotic human factors and controlled workflow structure. Unlike a purely algorithmic system, an agentic LLM is capable of understanding and responding sensibly to unstructured human information like “Dr. Lee told me she is leaving at 2pm today,” or “I have four cancer cases in my queue already. Can you see if someone else is available to take this lung wedge Dr. Johnson submitted?”

The same structure also creates a practical escalation pathway. The department can define thresholds for automatic action, such as routine redistribution within a subspecialty pool when total weighted workload remains balanced, and thresholds for human approval, such as exceeding a pathologist's maximum weighted daily load, assigning outside a credentialed service, or changing a case already opened by a pathologist. In those situations, the agent would not silently move work. Instead, it would summarize the current state, explain the reason for escalation, propose one or more options, and ask a human to approve or revise the plan. The result is an agentic workflow that maintains most of the benefits of deterministic workload rules, while also incorporating live operational context, natural-language interaction, and selective human input.

#### 2.4.3 Example 3: Real-time decision support

A third example is a complex clinical decision support scenario: ordering special stains during workup of a malignancy. In the previous reflex testing example, the key decision pathway is comparatively rigid: if a new colorectal carcinoma is diagnosed and MMR IHC has not been reported, the next step is to order MMR IHC as instructed by local policy. By contrast, in this example, the ancillary test recommendations are not predetermined. The pathologist may be evaluating an undifferentiated tumor, ruling out a possible infection, or attempting to resolve a specific diagnostic question. Thus, the task for the agent in this example is not simply to confirm whether a fixed task has been completed, but to assist the pathologist in selecting a panel of stains to facilitate a diagnosis.

In this workflow, the pathologist initiates the agent by asking for help ordering stains from within the reporting environment. The agent reviews the current report draft, specimen type, gross description, clinical history, prior pathology diagnoses, relevant laboratory or imaging context if available, and any stains already ordered or resulted. It then retrieves authoritative background information from approved sources, such as the department's own curated diagnostic knowledge base, the local IHC and histochemistry test menu, and selected external references indexed through a RAG system. The agent synthesizes the retrieved information and presents a list of recommendations that are 1) actually available for ordering in the local laboratory, and 2) tailored to the current diagnostic question, with each recommendation supported by a link to a specific item from the referenced knowledge bases. The pathologist can then accept the recommendations, decline the recommendations, or discuss potential modifications with the agent. Once the pathologist has made their decision, the agent places an order for the required stains and drafts a table to record the results in an appropriate section of the report.

In this example, a simplified case state record might include the draft diagnostic problem, the current differential diagnosis, the selected tissue blocks, stains already performed, and whether the pathologist has approved ordering:

{

“case_id”: “example-case-123”,

“current_state”: “assemble_case_context”,

“specimen source”: “liver”,

“specimen description”: “liver mass biopsy favor metastasis”,

“draft_diagnosis”: “poorly differentiated malignant neoplasm”,

“diagnostic_question”: “Select immunostains to determine tissue of origin and tumor type”,

“block_for_testing”: “A1”,

“stains_already_reported”: [“AE1/AE3”, “S100”, “MART-1”],

“proposed_stain_panel”: **null**,

“pathologist_approval”: **null**

}

The workflow template defines how the agent moves from context gathering to recommendation, approval, ordering, and optional interpretation support. Ordering and result interpretation remain under pathologist control:

{

“assemble_case_context”: {

“context_complete”: “retrieve_reference_support”,

“context_incomplete”: “request_pathologist_clarification”

},

“retrieve_reference_support”: {

“references_retrieved”: “check_local_test_menu”,

“references_conflicting”: “summarize_uncertainty_for_review”

},

“check_local_test_menu”: {

“recommended_tests_available”: “propose_stain_panel”,

“recommended_tests_unavailable”: “propose_available_alternatives”

},

“propose_stain_panel”: {

“pathologist_approves”: “order_approved_stains”,

“pathologist_modifies”: “order_modified_panel”,

“pathologist_rejects”: “record_no_order”

},

“order_approved_stains”: {

“orders_placed”: “await_stain_results”

},

“await_stain_results”: {

“results_available”: “support_result_interpretation”

},

“support_result_interpretation”: {

“interpretation_complete”: “return_to_pathologist_report_editing”,

“pattern_uncertain”: “summarize_uncertainty_for_review”

}

}

As in the previous examples, as the agent traverses this workflow it uses tools. A report-context tool retrieves the draft diagnosis, microscopic description, specimen site, block information, and stains already resulted. A history tool retrieves prior pathology diagnoses, relevant prior ancillary studies, and clinical history information. A retrieval tool searches the department's curated diagnostic knowledge base and vetted external references for stains relevant to the diagnostic problem. A test-menu tool filters the candidate stains against what the lab can offer, preventing the agent from recommending markers that are unavailable. An ordering tool places orders for the stains approved by the pathologist, on the selected block, with the same audit trail and permissions that would apply to a manual order.

Importantly, in this system the recommendations presented to the pathologist need not be a “black box.” The ideal output would be a short, source-linked recommendation: for example, a first-line panel to establish broad lineage, additional markers to evaluate specific diagnostic considerations raised by the clinical history, and a note that two literature-supported markers are not on the local test menu but have available local substitutes or require send-out. Each proposed stain could include a brief rationale, the question it addresses, relevant limitations, and links to the supporting references. The pathologist can approve the entire panel, remove unnecessary stains, add others, ask clarifying questions about why a stain was suggested, or ask the agent to narrow the panel to conserve tissue.

This workflow differs from conventional clinical decision support pathways in a few important ways. First, the decision-support tool has access to live case context, rather than operating as a standalone search or lookup. The agent might see, for example, that the report draft describes a poorly differentiated malignant neoplasm in a liver biopsy, that the patient has a remote history of melanoma, that a cytokeratin AE1/AE3 stain is already positive, and that the specimen has one available tissue block. Second, the recommendations are constrained by local operational reality. The agent checks the lab test menu, evaluates block availability, and reviews any relevant local rules before proposing the ordering plan. Third, the interaction with the agent is conversational and iterative: The pathologist can ask, “Does your panel distinguish ovarian origin?” or say, “Give me the smallest panel that reliably separates cholangiocarcinoma from metastatic lung cancer,” and the agent can translate that request into a structured, evidence-linked recommendation.

The agentic structure is also useful after the results are obtained. If the pathologist asks for help interpreting a pattern, the agent can retrieve the original diagnostic question, the stains ordered, the staining results, the clinical history, and the same reference sources used to recommend the panel. It can then provide interpretive aid, such as a table of which diagnostic considerations are supported or weakened by the observed pattern.

## 3 Additional agentic AI use cases in anatomic pathology

In this section and in Table 1, we elaborate other potential uses of agentic systems in anatomic pathology, but note that these uses are merely practical extensions of the agentic workflow patterns described in the examples above: access to local systems, workflow rules, state tracking, retrieval of external data, access to software tools, audit functionality, and escalation for human review where clinical judgment or institutional authority is required.

**Table 1:** *Example use cases.* A non-exhaustive list of potential applications for agentic AI in anatomic pathology. In these workflows, an agent coordinates tools according to task-specific instructions and fixed safeguards.

Use case	Starts when	Most consequential action	Main risk	Main human checkpoint
*Diagnostic workflow*				
Summarize clinical and pathology history	Pathologist opens a case	Reads records	Omits or misrepresents important history	Confirm important findings against original sources
Reflex testing support	Draft diagnosis is entered	Places an order after human approval	Misclassifies a diagnosis so the wrong reflex test is suggested	Confirm the machine-interpreted diagnosis and the proposed reflex test before ordering
Ancillary test decision support	Manual trigger by pathologist during case workup	Places an order after explicit approval	Recommends a plausible but incorrect panel	Review each proposed ancillary test order and its rationale
Synoptic report preparation	Draft diagnosis is entered	Adds structured data to a draft report	Populates a field with information not supported by the narrative diagnosis	Review every populated field against the narrative diagnosis before sign-out
*Quality and safety*				
Report proofreading during drafting	A draft is saved	Flags errors and suggests edits; changes nothing	False positives cause alert fatigue	Adjudicate and approve every proposed edit
Significant diagnosis communication	A case is signed out	Sends a message outside the department	Sends the message to the wrong person or omits required information	Approve recipient and message
Turnaround time monitoring	Runs on a schedule	Reads case status and sends status updates	Misses a delayed case or includes unnecessary information in an update	Verify case status and proposed message before approving an update
*Practice management*				
Workload balancing and case routing	Runs continuously	Assigns cases using defined workload balancing and prioritization criteria	Uses outdated availability information or violates staffing constraints	Approve exception requests and rejected assignments
Consultation and tumor board	Manually triggered	Sends packet with case material links, relevant history, and diagnostic questions	Prepares an incomplete or incorrect information packet	Approve the packet and recipient list

### 3.1 Diagnostic workflow optimization

The most immediate anatomic pathology use cases for agentic systems are likely to occur inside the diagnostic workflow but strictly outside the act of rendering a diagnosis. Many such tasks begin with context gathering: Before sign-out, an agent could retrieve prior pathology reports, recent procedures, relevant imaging impressions, molecular results, cytology material, and prior diagnoses. Rather than forcing the pathologist to search multiple parts of the record, the system could present a compact, source-linked summary with accession numbers, dates, and direct links back to the original material. Prior work on LLM-based pathology summarization and structured extraction supports the feasibility of these component tasks, though clinical deployment would require local validation and careful attention to error modes.^30^, ^33^, ^36^

Agentic workflows could also help with follow-up actions that are currently easy to miss or that add delay. As described in the first example above, an agent could detect that a report lacks required reflex testing, verify whether testing has already been ordered or resulted, apply the local reflex testing policy, and prepare the next recommended action for approval by the pathologist. In another workflow, the system could recognize that a report requires a synoptic template, retrieve the appropriate reporting elements from the narrative draft, and prepopulate synoptic template fields. Studies of LLM-based synoptic reporting and report classification, and of vision-language-model-based pathology report generation, support their feasibility, and an agentic system can connect those operations to local rules, source systems, and review steps.^35^, ^40^, ^78^

A third opportunity is communication support. For example, the College of American Pathologists recommends that institutions define policies for communicating significant or unexpected diagnoses in anatomic pathology.^79^ With an understanding of clinical context enabled by the workflows described previously, an agent could identify cases likely to merit communication, identify the appropriate clinician, prepare a draft message that includes the diagnosis or other relevant information, then route the message to the pathologist for editing and approval before sending to the clinician. The same pattern could support drafting updates to clinical submitters for cases delayed by pending ancillary studies, or could support consultation workflows by assembling the case history, relevant previous results, pending stains, and by drafting a cover letter.

### 3.2 Quality and safety

Quality and safety workflows are good candidates for agentic AI assistance because they require vigilance across many small details and/or constitute tasks that, though they are relatively simple and repetitive, are time-consuming and require judgment and understanding. One area of opportunity is in-flight proofreading and quality review of diagnostic reports in preparation. For example, an agent could review a draft report for typographical errors, internal inconsistencies, missing synoptic elements, laterality conflicts, discordance between the diagnosis and other report elements such as immunostain results. The agent reviews the report contents against pathology history for pitfalls; for example, if a prior biopsy and current resection appear discordant, it asks the pathologist whether the discordance is intentional, perhaps explained by treatment effect, or is worth addressing in a comment. The agent also monitors the clinical history field and other requisition sections for special requests. For example, if the requisition states that tissue was obtained for tumor profiling, but no tumor profiling order has been placed and the request has not been addressed in the draft report, the system flags the discrepancy before sign-out. These tasks are feasible for LLMs. GPT-4 has been evaluated for automated detection of deliberately introduced errors in oncology pathology reports, with substantial error-detection performance but also a higher false-positive rate than expert human review, highlighting both the opportunity and the need for human oversight.^37^

Quality assurance and patient safety opportunities extend beyond the individual report. Agentic workflows could monitor queues for overdue reports or cases with delayed ancillary studies, escalating them for human intervention. They could also support systematic quality improvement efforts by extracting structured information from report archives to surface important diagnostic trends such as rates of indeterminate diagnostic categories over time.^80^, ^81^, ^82^ The agent's contribution could be both the scheduled classification of narrative diagnostic text into discrete categories suitable for analysis, and the continuous surveillance of reporting trends. Prior work on operationalizing LLMs for clinical research data extraction and extraction of structured data from pathology reports supports the viability of these ideas.^17^, ^31^, ^34^

### 3.3 Practice management

Use of agentic systems for administrative tasks is a natural extension of existing interest in using language models for coding, prior authorization, and quality-measure reporting, where the value lies in reducing administrative friction.^14^, ^15^, ^16^ Daily practice management in pathology is filled with small coordination tasks that require mundane but time-consuming human effort and contextual understanding, but not diagnostic judgment. Agentic workflows could potentially help departments coordinate this administrative work, and they could be especially useful where relevant information is scattered across multiple channels like spreadsheets, shared documents, LIS data, EHR data, or digital pathology system data. The second example above described how a workload balancing and case routing agent could monitor the day's accession mix, retrieve relevant staffing and subspecialty rules, identify likely bottlenecks, and assist with distribution of work. Similar workflows could support consultation routing, case triage, tumor board preparation, and the tracking of cases awaiting outside ancillary testing or consultation.

A particularly interesting opportunity arises where agentic LLM capabilities are paired with diagnostic AI outputs and operational metadata. For example, a digital pathology algorithm might identify cases likely to contain malignancy or identify other features that influence the work effort and priority of a case. An agentic workflow could use those diagnostic results together with specimen type, due date, pathologist availability, and service rules to prioritize and assign cases to balance workload and to optimize turnaround times.

## 4 Discussion

Across the aforementioned examples of practical agentic AI deployment in anatomic pathology, the agent's principal value derives from orchestrating workflows where it maintains awareness of current state, proposes or executes the next action in looping format, and escalates for human intervention where appropriate. The opportunities presented here raise important questions about how such systems could be implemented, and about how to ensure that they support useful, safe, auditable, and locally adaptable workflows.

### 4.1 Practical considerations

#### 4.1.1 Integration strategies

The LIS integration landscape is perennially challenging, with persistent barriers around data compatibility, security, evolving standards, validation, and post-implementation modification.^83^ AI integration projects can be expected to meet many of the same roadblocks.

In our experience, few LISs provide comprehensive APIs, and even those that do may not expose every data element, interface message, work-queue state, assignment rule, data dictionary, order capability, or other data that could be useful to the designer of an agentic system. Thus, a likely near-term implementation model is for LIS vendors to embed agentic functions directly within their proprietary systems. This approach has obvious advantages. The agent can operate close to native software workflow states using the vendor's existing security and audit infrastructure, and interact with accessioning, result entry, case assignment, report text, and case metadata without requiring an external interface for every action. The drawback is that vendor-controlled agents may also be narrow, opaque, and slow to adapt to local needs. If each vendor implements agentic functionality only inside its own platform, labs may gain some conveniences while losing the ability to build workflows that reflect local practice rather than generic vendor product designs.

A second model is an interoperable, API-first approach in which LIS vendors expose well-documented, safe interfaces that allow customers to build their own agents. For anatomic pathology, a standards-based API-first model is attractive because the most useful workflows are often local.^84^, ^85^ A tertiary academic practice, a community hospital practice, and a multi-site commercial lab probably have different workload management patterns, subspecialty structures, ancillary testing rules, report templates, staffing constraints, and business priorities. Custom agents tailored to each setting could use the same APIs to do the work of agentic AI while respecting each organization's local priorities and capabilities. This same API-first approach would also allow labs to connect their agents to non-uniform, non-LIS data sources, such as scheduling systems, local knowledge bases, digital pathology platforms, and institutional policy libraries.

Despite its advantages, an API-first approach based on industry standards is not without substantial challenges. FHIR provides a standards-based framework that could facilitate agentic workflows, but standards-based APIs have been implemented unevenly across healthcare systems, and their integration into clinical workflows remains challenging.^83^, ^84^, ^85^ Pathology workflows often rely on specialized local configurations of work queues, case and task states, rule logic, dictionaries, and other configurations that available standards-based API resources and profiles may not expose completely or consistently to external tools.^86^, ^87^, ^88^

Thus, the ideal state for labs is probably not a choice between proprietary vendor agents and customer-built agents. Instead, it is both. Vendors should provide safe baseline agentic functionality for labs that cannot or do not want to build their own systems, using standardized input formats and structured mechanisms for bringing local organizational data into the vendor environment. At the same time, vendors should expose explicit, well-documented interfaces, so more technically mature organizations can build and validate their own agents. In that hybrid model, the vendor supplies dependable default capabilities and standard execution paths, whereas customers retain the ability to innovate where local variation is important.

Even under ideal conditions, configuring advanced agentic workflows may require complex architectures pieced together using APIs, traditional interface engines, local middleware, or vendor-specific software adapters.^83^, ^89^ Each component application may require institutional authentication controls, API gateways, interface engine feeds, secure storage and compute infrastructure, customized network configurations, and other supporting functionalities. Configuring such complex architecture requires substantial resources and coordination between lab operations, information technology, security, compliance, and vendor teams. The ensuing complexity creates financial, operational, validation, and maintenance barriers that may impede implementation, even if all agree that an agentic system is worth building.

#### 4.1.2 System life cycles

Prior work has emphasized that safe LLM adoption in pathology requires organizational preparedness, including governance, validations, privacy and security controls, education, and integration with existing LIS and EHR infrastructure.^90^, ^91^ Agentic workflows intensify these requirements because they may couple LLM reasoning to real-world actions in addition to textual outputs.

Organizational preparedness for agentic AI deployment should be treated as an ongoing and iterative program rather than a one-time technical project. The National Institute of Standards and Technology AI Risk Management Framework organizes this process around four interrelated functions: Govern, Map, Measure, and Manage.^92^ Applied to a lab, these functions might include clearly defining the intended use, risk tolerance, accountable owners, and human-oversight responsibilities for the agentic system, documenting the workflow, data sources, users, and consequences of system failure, safely validating system performance under conditions representative of the intended deployment environment, and establishing ongoing monitoring, incident response, change management, and then performing periodic reassessment of the system.^90^, ^91^, ^93^

For lab applications, the Govern and Map functions might include convening formal governance to assign clear accountability for agentic AI system selection, local configurations, access approvals and controls, validation plans, user training, monitoring, incident response, revalidation after changes, and other tasks. Each agentic AI project should include a clear articulation of the system's intended use, authorized users, the boundaries of its authority, any foreseeable harms, conditions for validation and deployment, and any requirements for institutional audits or other external controls. Practically speaking, such controls should be proportional to the foreseeable harms. For example, a periodic retrospective audit alone may suffice for an agent providing low-risk administrative support, but agents that can alter reports, initiate orders, or take other consequential actions might require an elaborate set of controls and audits such as regular collection and analysis of performance data, regular and formal periodic approval of system performance by an accountable professional, and regular testing of safety and rollback mechanisms. This approach follows the NIST AI Risk Management Framework and current Joint Commission Coalition for Health AI guidance, both of which treat governance as continuing lifecycle oversight rather than a one-time project.^92^, ^94^

In the United States, regulation of AI in clinical systems is a complex and rapidly evolving subject.^95^ Whether and how a healthcare AI system is regulated depends on what the software does and how it is intended to be used, rather than simply on whether it uses AI. Agentic AI functions limited to administrative support of lab workflows would typically fall outside the FDA definition of a medical device, as would certain clinical decision-support systems that provide recommendations rather than direct clinical decisions, that allow the clinician to independently review the basis for a recommendation and exercise independent judgment, and that do not analyze medical images or device-generated signals.^96^, ^97^, ^98^ However, note that a permissive regulatory classification does not eliminate the lab's responsibility for local validation, change control, and continued quality oversight.^99^, ^100^ Medicolegal liability for AI errors is a similarly complex and evolving subject. Liability may extend to clinicians, healthcare institutions, developers, and vendors under various legal theories, depending on the source of the error, degree of autonomy, deployment decisions, and the manner of use.^101^, ^102^, ^103^

In general, validation (the Measure function) of agentic systems should start with testing of individual component performance on measures such as information retrieval performance, text extraction and classification accuracy, tool selection, adherence to task-specific instructions, and maintenance of accurate representations of workflow states. Recent publications provide a foundation for validating AI systems for clinical lab application.^99^, ^100^ These principles are also applicable to relevant components of agentic AI systems. Validation of the agentic layer itself should also occur: planning, tool use, authorization boundaries, multi-step execution, memory, escalation, and failure and recovery rates should all be tested. This phase should comprise end-to-end testing using simulated or segregated system data that simulates a complex clinical environment.^104^ End-to-end test trials should include uncommon or deliberately contradictory situations, induced system component failures, interrupted workflows, and other induced faults in order to evaluate system performance against known failure modes. During agent validation, system state information such as task state transitions, tool calls, proposed actions, escalation decisions, and error states should be captured for review. Appropriate validation performance metrics for agentic systems depend on the application, but in general, useful measures might include rates of task completion, inappropriate escalations or failures to escalate, erroneous proposed actions, unnecessary tool calls or tool failures, rates of human override of agent recommendations, and recovery after task interruptions or induced system faults, as well as performance metrics related to the agent's operational or clinical purpose.

After validation, a period of prospective silent-mode evaluation can be useful in assessing the configured system against live workflow conditions while preventing its outputs from influencing important decisions or altering clinical state.^105^, ^106^ Under ideal conditions, human factors testing also can be used to determine whether users understand the system and its controls and to determine whether the system is susceptible to automation bias, alert fatigue, or merely seems to add to the workload.^107^ Only after validation and testing success criteria are met should deployment progress to a real-world pilot with explicit human approval, limited privileges, and a tested fallback pathway, before full deployment. Post-deployment monitoring should evaluate for changes in performance characteristics such as error rates, human approval rates, tool use patterns, and system errors (the Manage function). As with other clinical systems, modifications to models, prompts, task instructions, knowledge sources, interfaces, permissions, or other system components should trigger consideration of revalidation.^100^

#### 4.1.3 Safety and security

Agentic systems introduce security and safety risks that may differ from those of the standard software tools used by labs. Agents may have access to protected health and confidential business information, and they may also have access to powerful tools such as messaging systems and clinical ordering functions as well as to external documents. Malfunction of an agent with excessive functionality, excessive permissions, or excessive autonomy can cause serious damage.^108^

Agentic systems are vulnerable to errors induced by incorrect or stale information or by malicious content that can “poison” the model's context, causing reasoning failures.^109^ Long workflows may exceed a model's context window, and even correct instructions or evidence retained within long contexts may not be used reliably.^110^, ^111^ Faulty reasoning occurring early in an agentic process may be treated as a valid premise for later decisions, allowing uncertainty and errors to propagate across subsequent steps.^112^, ^113^ Safe designs thus include deterministic and behavioral safeguards that verify high-consequence facts before actions, double-check workflow rules before important decisions, prevent duplicate actions and runaway processes, and periodically reconcile system state against authoritative source data. In general, processes where missing, contradictory, or stale results cannot be automatically repaired should trigger an explicit failed status requiring human review, rather than permitting the model to engage in unconstrained improvisation.

It is important to recognize that merely requiring a human approval click to advance an automated process does not by itself establish meaningful oversight. If the machine proposal is difficult to inspect or understand, or if the reviewer lacks the time or authority to meaningfully engage with or challenge the machine recommendation, then the human click is no more than theater. System designers should be aware of automation bias, a cognitive bias in which people tend to excessively trust automated systems.^114^ We recommend that human review checkpoints in agentic systems be designed so that machine proposals are presented in a comprehensible format, including all available information that a human reviewer may need to make an informed decision. For consequential decisions, the default response choice should be to deny approval. Continuous monitoring should include approval rates; checkpoints with very high rates of human approval may be more fully automatable, thereby reducing alert fatigue, whereas processes with very low rates of approval may benefit from redesign.

Agentic systems using language models are also vulnerable to adversarial actions such as prompt injection attacks.^115^ In a prompt injection attack, malicious instructions embedded in case text, documents, messages, or retrieved web content can be viewed by the model as legitimate instructions, which can cause the model to attempt destructive actions such as exfiltration of private data. A complete review of agentic LLM security best practices is beyond the scope of this manuscript, but in general, practical safeguards include: least-privilege permissions, clear separation between read-only and write-capable tools, explicit thresholds for human approval of consequential actions, clear presentation of data source provenance, audit logging, rollback pathways, and clear separation of untrusted content from executable instructions provided to the model.^94^, ^116^

Finally, despite the current enthusiasm for agentic AI implementation across many industries including healthcare,^117^ it is important to remember that agentic AI is not the best tool for every task. In essence, LLMs are statistical models that predict the most likely continuation of a given sequence based on their training data. Sometimes, this probabilistic approach results in plausible appearing but incorrect outputs, a phenomenon called “hallucination.”^118^ In addition, the same input can produce different outputs on repeated runs of an LLM, because LLMs generate text by probabilistically selecting each successive output segment. Further, behavioral instructions provided to an agent in natural language may be ambiguous or imprecise when compared with the explicit logic and deterministic control structures imposed by formal programming languages. For these reasons, many workflows with stable inputs, clear decision pathways, and fixed rules can often be implemented more safely, predictably, and economically using ordinary rule-based algorithms. Agentic architecture is most appropriate when inputs are heterogeneous or unstructured, when appropriate next actions may not be completely specified in advance, or when systems must understand and respond sensibly to unpredictable inputs. Even then, most real-world agentic systems are best conceived as hybrid systems in which agentic systems mediate between an array of deterministic tools and operate within a clear set of software “guardrails” for agent actions. Deterministic rules are especially useful for high-stakes modules such as security and safety, where reliance on behavioral instructions alone may be insufficient.^119^

#### 4.1.4 Cost and sustainability

Institutions must also prepare for the cost of agentic systems, which could be substantial. A recent analysis of commercial LLM API spending estimated that expenditures more than doubled from $3.5 billion in late 2024 to $8.4 billion by mid-2025.^120^ LLM-based agentic workflows are particularly resource-intensive, with repeated LLM calls for planning, retrieval, tool selection, output generation, verification, and logging, and those calls may include very long prompts containing report text, prior history, local policies, and structured case data.

Commercial LLM APIs provide state-of-the-art models and reduce local infrastructure needs, but they create recurring usage costs and require acceptable contractual controls for privacy, security, retention, availability, and model changes. Commercial LLM APIs are typically billed by input and output volume, so costs will scale with prompt length, response length, number of agent steps, and the number of items processed. Conversations and strategy sessions for how to reduce LLM costs will likely become commonplace.^121^ Deployment using local models can reduce API spending, but shifts costs toward hardware, infrastructure, maintenance, and technical staffing.^33^, ^122^ Local model performance relative to commercial models is model- and task-dependent. Smaller local models may underperform frontier commercial models on some tasks, whereas frontier open models achieve comparable performance in others.^123^, ^124^ A hybrid architecture may be practical, with local models handling simple or sensitive data and external models reserved for specific approved tasks, though this approach increases the number of boundaries that must be secured, monitored, and validated.^33^

Finally, large-scale LLM use has environmental costs, including energy and water use, that should be considered as part of responsible deployment.^125^ Larger models may improve results for some tasks, but they also increase computation, cost, energy use, and natural resource consumption.^125^ Labs should therefore ordinarily select the smallest model and simplest workflow that meet performance requirements, avoid unnecessary model calls and repeated processing, and consider environmental burden alongside cost, latency, and accuracy when comparing deployment options.

As described in the preceding discussions on integration and system life cycles, agentic AI system costs also include the less visible work of integration, validation, monitoring, security review, governance, training, testing, and ongoing management; experience from radiology AI integration suggests that custom integrations can create substantial operational and maintenance burden.^89^ For labs, the practical implication is that agentic AI should be evaluated as a potentially sizable investment that must deliver proportionate benefits.

Because of these risks and costs, in addition to their considerable technical complexity, agentic systems may be better suited to incremental deployment rather than installation as a single transformative monolith. For example, adoption could begin with a few simple and constrained workflows, such as retrieving relevant clinical history or monitoring a queue for delayed cases. Each subsequent additive step can then be individually scoped, validated, monitored, and revised as the system is allowed to take on a broader set of actions. Incremental development and deployment allow labs to build trust, to define and prepare for failure modes, to establish good safety and security practices, and to prepare to accommodate system costs while steadily moving toward more capable systems.

### 4.2 Future design patterns

As agentic systems mature, some workflows may involve multiple specialized agents rather than a single agent with every capability. For example, a pathologist-facing agent might coordinate with a departmental scheduling agent, a lab operations agent, or a vendor-hosted LIS agent. Google's Agent2Agent protocol has been proposed as an open standard for allowing agents built on different frameworks or vendor platforms to communicate, securely exchange information, and coordinate actions across enterprise systems.^126^ Multi-modal models create another path forward by allowing the same agentic system to integrate report text, clinical context, and whole-slide image data. Prior work has demonstrated pathology-specific question answering and report generation from whole-slide image inputs.^41^, ^78^ An agentic system could integrate such models as tools, creating very powerful and comprehensive end-to-end pathology workflows.

### 4.3 Current state of the industry

There is active vendor interest in LLM integration into commercial pathology software, with a small contingent of pathology-related products promising features like report drafting, extraction of requisition data, report proofreading, clinical decision support, workload balancing, and coding support.^127^, ^128^, ^129^ Vendors have begun to announce products that integrate digital pathology and LLM-enabled workflows,^130^ and activity around explicitly agentic pathology workflows is also beginning to appear in the marketplace.^127^ Major cloud providers have taken an interest; Amazon Web Services has published an agentic digital-pathology reference architecture using report-generation and report-validation agents.^131^ These early offerings suggest that vendors see anatomic pathology as a near-term target not only for LLM-assisted reporting but also for case orchestration and workflow automation.

### 4.4 Limitations

This article is a conceptual narrative review and synopsis of a complex and rapidly evolving aspect of the healthcare landscape. The provided examples illustrate plausible architectures and control patterns, but they are merely sketches that sacrifice completeness for clarity. Real-world designs would be far more complex, particularly with regard to exception and error handling, and would need to accommodate myriad real-world human preferences and operational constraints. Real systems may encounter ambiguity and incomplete or incorrect information that could place a workflow into an unresolved state, so real-world systems must account for these error conditions, and should provide pathways for graceful resolution such as reverification of source information, re-evaluation of existing data, and/or escalation for human review before a faulty agentic workflow is permitted to advance.

## 5 Conclusion

The pace of clinical AI adoption has lagged behind some optimistic public speculation.^132^, ^133^ The prospect of transformative cost savings and efficiency gains from agentic AI has unsurprisingly generated a massive amount of interest.^134^ Recently, public comments from a prominent health care leader speculating about a reduced need for radiologists—if only regulatory barriers could be addressed—were reported in the mainstream press.^135^ Some of this bullishness is warranted; the diagnostic specialties do involve repetitive pattern recognition and computational tools can change the economics of how that work is performed. However, an overly reductionist view of what diagnostic physicians do should be viewed as a pitfall for administrators. Even at a remove from the bedside, creative problem-solving, open communication, emotional intelligence, nuanced judgment, compassion, and the irreducibly human act of caring are important. Economic forces will continue to push healthcare organizations toward automation, but the human costs of this transformation should be understood.

In radiology, where AI products are more mature and numerous than in pathology, peer-reviewed evaluations have found that some commercially available diagnostic AI products lack peer-reviewed evidence, that most supporting studies emphasize diagnostic accuracy rather than actual clinical outcomes, and, importantly, that real-world deployment requires (and can be delayed by) local validation, interoperability with PACS/RIS and EHR workflows, monitoring, governance, and management of clinical responsibilities beyond image classification.^89^, ^136^, ^137^, ^138^ Anatomic pathology shares many of the same challenges as radiology, so it is reasonable to expect a similarly prolonged and uneven adoption curve for pathology.

Agentic AI may follow a different, and potentially faster, adoption path than diagnostic AI algorithms. The practical near-term value of agentic AI systems in anatomic pathology is not in replacing diagnostic judgment, but in orchestrating the information work around the judgment: gathering clinical context, triaging complicated inputs, preparing draft outputs, monitoring quality signals, and reducing workflow friction. As described above, because these applications are generally considered to be workflow support, documentation assistance, quality surveillance, or decision support rather than autonomous diagnosis, they will face a different and probably less rigorous scrutiny on safety and regulatory grounds. That does not make them risk-free; they still require validation, governance, monitoring, auditability, and clear human oversight—but it does remove important barriers.

In that sense, agentic AI offers a plausible and constructive near-term vision for AI in pathology: not the replacement of pathologists, but better-supported diagnosis that allows pathologists to spend more of their time on the judgment, accountability, and genuine care that remain distinctly human.

## Declaration of competing interest

The authors declare that they have no known competing financial interests or personal relationships that could have appeared to influence the work reported in this article.
